# Effects of Exogenous Androgens on Platelet Activity and Their Thrombogenic Potential in Supraphysiological Administration: A Literature Review

**DOI:** 10.3390/jcm10010147

**Published:** 2021-01-04

**Authors:** Adrian Eugen Roşca, Ana-Maria Vlădăreanu, Alina Mititelu, Bogdan Ovidiu Popescu, Corin Badiu, Constantin Căruntu, Suzana Elena Voiculescu, Minodora Onisâi, Şerban Gologan, Radu Mirica, Leon Zăgrean

**Affiliations:** 1 Division of Physiology and Neuroscience, Department of Functional Sciences, Carol Davila University of Medicine and Pharmacy, 050474 Bucharest, Romania; suzana.voiculescu@umfcd.ro (S.E.V.); leon.zagrean@umfcd.ro (L.Z.); 2 Victor Babeş National Institute of Research-Development in the Pathology Domain, 050096 Bucharest, Romania; bogdan_ovidiu_popescu@yahoo.com; 3 Department of Cardiology, Emergency University Hospital of Bucharest, 050098 Bucharest, Romania; 4 Department of Hematology, Carol Davila University of Medicine and Pharmacy, Emergency University Hospital of Bucharest, 050098 Bucharest, Romania; ahmititelu@yahoo.com (A.M.); minodorel@yahoo.com (M.O.); 5 Department of Neurology, Carol Davila University of Medicine and Pharmacy, Colentina Clinical Hospital, 020125 Bucharest, Romania; 6 Department of Endocrinology, Carol Davila University of Medicine and Pharmacy, C.I. Parhon National Institute of Endocrinology, 11863 Bucharest, Romania; badicrin@yahoo.co.uk; 7 Division of Physiology, Department of Fundamental Disciplines, Carol Davila University of Medicine and Pharmacy, 050474 Bucharest, Romania; costin.caruntu@gmail.com; 8 Department of Dermatology, “Prof. N.C. Paulescu” National Institute of Diabetes, Nutrition and Metabolic Diseases, 011233 Bucharest, Romania; 9 Department of Gastroenterology, Carol Davila University of Medicine and Pharmacy, Elias Clinical Hospital, 011461 Bucharest, Romania; serbangologan@gmail.com; 10 Department of Surgery, Carol Davila University of Medicine and Pharmacy, “Sf. Ioan” Clinical Hospital, 042122 Bucharest, Romania; mirica_rm@yahoo.com

**Keywords:** anabolic androgenic steroids, AAS, androgens, testosterone, hemostasis/haemostasis, platelet activity, platelet reactivity, platelet aggregation, thrombopoiesis, platelet count, prothrombotic state, thrombotic diathesis, thrombosis

## Abstract

Anabolic androgenic steroids (AAS), simply called “androgens”, represent the most widespread drugs used to enhance performance and appearance in a sporting environment. High-dosage and/or long-term AAS administration has been associated frequently with significant alterations in the cardiovascular system, some of these with severe endpoints. The induction of a prothrombotic state is probably the most life-threatening consequence, suggested by numerous case reports in AAS-abusing athletes, and by a considerable number of human and animal studies assessing the influence of exogenous androgens on hemostasis. Despite over fifty years of research, data regarding the thrombogenic potential of exogenous androgens are still scarce. The main reason is the limited possibility of conducting human prospective studies. However, human observational studies conducted in athletes or patients, in vitro human studies, and animal experiments have pointed out that androgens in supraphysiological doses induce enhanced platelet activity and thrombopoiesis, leading to increased platelet aggregation. If this tendency overlaps previously existing coagulation and/or fibrinolysis dysfunctions, it may lead to a thrombotic diathesis, which could explain the multitude of thromboembolic events reported in the AAS-abusing population. The influence of androgen excess on the platelet activity and fluid–coagulant balance remains a subject of debate, urging for supplementary studies in order to clarify the effects on hemostasis, and to provide new compelling evidence for their claimed thrombogenic potential.

## 1. Introduction

Anabolic androgenic steroids (AAS) are synthetic derivatives of testosterone, primarily designed to have both an enhanced anabolic and a reduced androgenic activity compared to the parent molecule [1,2]. The anabolic effect is based on their ability to stimulate protein synthesis, muscle mass growth and erythropoiesis. Over 100 synthetic substances have been introduced in clinical practice as treatments for various conditions, such as hypogonadism, anemia secondary to bone marrow insufficiency or renal failure, hereditary angioedema, osteoporosis, catabolic states associated with HIV infection, cancer, burns, chronic obstructive pulmonary disease, alcoholic hepatitis, or neuromuscular diseases [3,4,5].

Their powerful anabolic properties have led to an illicit use, with them being currently abused in high doses by athletes for enhanced performance, or in the bodybuilding community for cosmetic reasons [5,6,7]. Even if AAS have a strong anabolic activity, that mimics natural steroids, they still possess androgenic activity [8,9] (Table 1). AAS misuse produces both physical and psychological dependence [6,10,11], leading to long-term self-administration in progressively higher doses and stronger drug combinations [10]. Athletes use two or three different substances simultaneously (stacking), with a total dose equivalent of 600–1000 mg testosterone per week, sometimes up to 3000–5000 mg per week. This equals a blood concentration of 10 to 100 times higher than the physiological one [12]. For instance, considering a parenteral dose of testosterone enanthate or cypionate of 150–200 mg every two week in men with hypogonadism [13], the aforementioned abus dose is 6 to 500 times higher than that recommended in testosterone replacement therapy. AAS abuse is widespread and extending, becoming an alarming public health issue [10,14,15].

The negative impact of AAS excess is a consequence of their numerous side effects, including endocrine dysfunctions, alterations of the cardiovascular system, liver toxicity, behavioral changes and even psychiatric disorders, some of them with severe endpoints [10,12,14,15,16,17]. The negative effects on the cardiovascular function have classically been divided into four categories: thrombotic, atherogenic, vasospastic, and cardiomyocyte direct toxicity [18]. AAS-induced cardiovascular events are mostly present in a young and healthy population, with no associated risk factors or proven atherosclerotic lesions [19,20,21,22,23,24]. This suggests that the pathophysiological mechanism might be atherothrombotic rather than atherogenic, or vasospastic. This conclusion seems more logical in the attempt to explain the basis for the severe adverse effects of androgens misuse [22,25,26].

This review covers the available literature data regarding the influence of androgens usage on platelet function and thrombopoiesis, focusing on their thrombogenic potential in supraphysiological administration, probably the most important cardiovascular side effect emerging from AAS abuse.

## 2. Overview of Thrombogenic Potential of Exogenous Androgens

The thrombogenic potential of androgens has become a matter of high interest as AAS abuse in the sports environment has led to an alarming rate of severe cardiovascular events [10,27,28]. A considerable number of case reports has been gathered up to date, beginning with the report of McNutt et al. (1988), who noted a myocardial infarction in a weightlifter following AAS abuse [20]. These depict a comprehensive picture of the thromboembolic potential of AAS in high dosages and/or long-term administration. The nonmedical use of these doping substances has been linked to coronary heart disease and myocardial infarction [22,29,30,31,32,33,34,35,36,37,38,39,40,41,42,43,44,45,46,47,48,49,50], intra-ventricular thrombosis [23,32,51], cerebrovascular events [38,52,53,54,55,56,57,58,59,60,61], peripheral arterial and deep venous thrombosis [51,55,62,63,64,65,66,67], pulmonary embolism [65,66,67,68], retinal vein branch occlusion [69], superior sagittal venous sinus thrombosis [53], abdominal arterial infarction [64], renal infarction [37,70,71], or even sudden death [19,24,46,72,73,74,75,76,77,78]. Several of the epidemiological studies with a follow-up period of 4 years minimum reported a cardiovascular mortality rate in men exposed to non-therapeutic AAS that was more than double compared to non-users, justifying all the measures taken to decrease AAS misuse among both competitive and amateur athletes [79,80].

The thrombogenic potential of androgens has also been noted in their clinical usage. Cerebral venous thrombosis caused by AAS administration was strongly suggested in a case-report of a 40-year-old woman with aplastic anemia treated with oxymetholone for two years [81]. Male hormones and protein-assimilating hormone therapy in 27 hypoplastic anemia patients resulted in three cases of sagittal sinus thrombosis [82]. A case of acute myocardial infarction was reported in a non-athlete who inadvertently received therapeutic intravenous testosterone for hypogonadism [83,84], as well as a case of stroke secondary to testosterone therapy in a 21-year-old hypogonadal man [84]. A meta-analysis of 27 placebo-controlled studies detected an increased risk of cardiovascular events in men who received testosterone therapy, compared to placebo [85]. A population-based case-control trial has recently reported an increased risk of venous thromboembolism after testosterone therapy, peaking within six months after the treatment cessation [86]. On the other hand, there are data indicating that low serum testosterone concentrations are also associated with increased cardiovascular risk and mortality, and that testosterone replacement therapy may have beneficial effects [87,88]. Several recent systematic reviews and meta-analyses did not find clear evidence of increased risk for cardiovascular events in men with testosterone prescription [89,90]. Moreover, low plasma androgen levels in hypogonadal patients have been linked to deficient platelet function, explaining the presence of hemorrhagic diathesis observed in this population [91,92].

Unfortunately, the human data evoking the thrombogenicity of AAS are inconclusive because the findings come mostly from case reports (which generally include a reduced number of individuals), a few epidemiological studies, and a small number of cross-sectional studies. The only appropriate “prospective” studies related to testosterone-induced thrombogenesis are the experimental ones using animal models of thrombosis, but even those are few. Uzunova et al. performed several in vivo experiments in the 1960–1970s [93,94,95]. Testosterone cypionate administered in a dose of 10 mg/kg, twice weekly for 2 or 4 weeks, resulted in a reduced obstruction time, increased thrombus weight and high mortality rate in mice, rats or rabbits subjected to occlusive arterial thrombosis, in response to chemical or mechanical stimuli. The administration of flutamide (an androgen receptor blocker), or aspirin (an antiplatelet drug, known as an inhibitor of prostaglandin synthesis), significantly reduced mortality in testosterone-treated animals. The author hypothesized that the thrombotic potential of androgen excess may be related to an increase in endoperoxides and thromboxane generation in platelets [93,94,95]. Later, Rosenblum et al. (1987) resumed the in vivo experiments of arterial thrombosis, showing a faster onset of aggregation in the mesenteric arterioles of the male mice following the implantation of pellets with either 1.0 mg testosterone or 0.1 mg dihydrotestosterone (DHT) [96]. However, this effect has been considered more likely to be secondary to the endocrine function of the endothelium or adjacent tissue than to the platelet’s activity itself. No effect of testosterone or DHT has been observed when the activation of platelet aggregation has been triggered in the mesenteric arterioles of female rats, nor in the pial arterioles of either sex [96]. Interestingly, at physiological doses, androgens seem to exert the opposite action, inhibiting the induced arterial thrombosis [97].

A high amount of evidence suggests that hemostatic disturbances may lead to cardiovascular disease (CVD), sometimes with severe thrombotic endpoints [98,99,100,101,102]. Direct proofs of androgens’ thrombogenic effects are scarce and come from animal models of thrombosis, but there is a considerable amount of indirect data emerging from studies that assess the influence of exogenous androgens on the key processes of hemostasis, such as platelet activation and aggregation (detailed in the following section of this article), or coagulation and fibrinolysis (recently and extensively reviewed) [28,103,104].

The thrombotic risk of supraphysiological androgens may also be indirectly linked to their ability to influence various cardiovascular risk markers. Exogenous androgens in high dosages and/or prolonged administration have been shown to be atherogenic, by inducing ample changes in the lipid profile (increasing low-density lipoprotein-LDL cholesterol, triglycerides and apolipoprotein-B, and decreasing high-density lipoprotein-HDL cholesterol and apolipoprotein-A_1_) [5,105,106,107,108,109], by increasing plasma homocysteine level [110,111], and by enhancing inflammation and oxidative stress at the endothelial level, with a consecutive reduction in NO synthesis [111,112,113,114]. AAS have also been shown to elevate blood pressure [111,112,115] and to induce hemorheological effects, through impairing vascular reactivity [105,111] and triggering a polycythemia-induced hyperviscosity syndrome (an intrinsic anabolic effect) [16,36,105]. Since the abovementioned altered parameters have been independently linked to various coagulation abnormalities (they lead to enhanced platelet adhesion and aggregation, activation of the coagulation cascade, and suppression of fibrinolysis [116,117,118,119,120,121,122,123,124,125,126,127]), it may be assumed that AAS abuse is prone to produce atherothrombotic phenomena also by influencing these cardiovascular risk markers. Moreover, the cardiotoxic effects of extraneous androgens and the structural damage to the heart muscle may lead to intracardiac thrombosis, also reported in athletes abusing AAS. Structural remodeling following myocardial injury, expressed by ventricular hypertrophy and the reparative process of fibrosis, is known to induce functional (electrical) remodeling and diastolic function impairment, thus causing an enhanced risk of arrhythmias onset and predisposing to intracavitary thrombosis occurrence, with subsequent systemic embolism [27,105,128,129,130,131,132].

## 3. Overview of Platelet Function

Platelets are key players in hemostasis and thrombosis, and are a contributing factor in cardiovascular diseases such as heart attacks and strokes. Their activity is related to their capacity to function under proper stimuli. Platelets have four major functions: adherence, activation and secretion, aggregation, and interaction with coagulation factors in order to form the hemostatic plug and maintain hemostasis.

In a fairly simplified view, the hemostatic activity can be considered as a sequence of cellular and molecular events delineated into overlapping phases. It starts with receptor–ligand bindings, leading to platelet activation through complex mechanisms mediated by intracellular signaling, calcium-dependent cytoskeletal changes, granules release and conformational changes of the fibrinogen (FBG) receptor α_IIb_β_3_ integrin (aka glycoprotein IIb/IIIa, GP IIb/IIIa), which supports platelet aggregation [133,134].

Primary platelet adhesion (1) is initiated by the binding of vWF (von Willebrand factor) to a specific glycoprotein (GP) from a circulating platelets’ surface, GP Ib/IX/V, and also to the subendothelial collagen. This transient “bridge” retains platelets in the injured area and facilitates the contact of other surface GP, GP VI with collagen. This further induces intracellular signaling, leading to platelet activation and the release of secondary platelet agonists, such as thromboxane A_2_ (TxA_2_) and adenosine diphosphate (ADP), from platelets’ dense granules. These soluble agonists, together with locally produced thrombin, contribute to platelet activation trough distinct but interconnected pathways [133]. In addition to its role as a platelet adhesion receptor, GP Ib/IX/V can mediate the intracellular signals, involving the phospholipase C (PLC) pathway and the TxA_2_ and ADP pathways, finally leading to the activation of integrin α_IIb_β_3_ [135]. GP VI is the major signaling receptor for collagen on the platelet surface. Platelet activation by GP VI is intermediated by the activation of PLC, ADP release from platelet dense granules (DG) and TxA2 synthesis from arachidonic acid [136].

Platelet activation (2) is a multistep process starting with multiple interconnected platelet receptor–ligand interactions. After adhesion, the soluble agonists thrombin, ADP, TxA_2_ and epinephrine are the primary drivers for platelet activation. Each of these agonists activates specific G protein-coupled receptors on the platelet surface, triggering intracellular signaling pathways, which is followed by integrin activation and increases in cytosolic Ca^2+^ (calcium) concentration, with subsequent cytoskeletal reorganization, FBG receptor activation through dimerization (GPIIb/IIIa), and granule secretion [133,137,138].

Phosphoinositide hydrolysis and eicosanoid synthesis pathways are representative of platelet activation.

The phosphoinositide hydrolysis (2a) pathway is initiated when ADP, TxA_2_ and thrombin bind to specific receptors on platelets’ surfaces, such as the following: purinergic receptor 2Y1 (P_2_Y_1_) for ADP; TP for TxA_2_; protease-activated receptor 1 and 4 (PAR_1_ and PAR_4_) for thrombin. This activates PLC, which hydrolyzes membrane phospholipids to form 1,4,5-inositol trisphosphate (IP_3_) and 1,2-diacylglycerol (DAG), which serve as second messengers. IP_3_ opens the Ca^2+^ channels in the platelet dense tubular system (DTS), which triggers Ca^2+^ influx through the plasma membrane. DAG activates protein kinase C isoforms in the presence of Ca^2+^, mediating platelet shape changes, platelet granule secretion, and also platelet aggregation [139,140]. Thrombin is the most powerful platelet activator, along with collagen. ADP, a weaker agonist, is released by the injured endothelial cells, as well as by DG upon platelet activation. It has two purinergic receptors, P_2_Y_1_ and P_2_Y_12_, both being required for the platelet ADP-induced response [141]. In platelets ADP induces or contributes to: shape change, granule release, TxA_2_ production, the activation of α_IIb_β_3_ integrin and subsequent platelet aggregation.

The eicosanoid synthesis pathway (2b) is initiated by the interaction of TxA_2_ with its receptor, which activates phospholipase A_2_ (PLA_2_). PLA_2_ is also activated by the rise in cytosolic Ca^2+^, and is potentiated by GP Ib/IX/V adhesion to collagen through vWF. PLA_2_ releases arachidonate from membrane phospholipids, which is converted by cyclooxygenase-1 to prostaglandins such as TxA_2_ [137,140]. TxA_2_ is an important agonist which contributes to the rise in cytosolic Ca^2+^, the activation of α_IIb_β_3_ integrin, and granule release. It diffuses across the plasma membrane along with other agonists (ADP) and contributes to the amplification of platelet activation and the recruitment of additional platelets.

The platelet inhibitory pathway (2c) represents a protective, inhibitory mechanism of platelet activation, which occurs via NO and prostaglandin I_2_ (PgI_2_). PgI_2_ stimulates its receptor (IP), inducing a rise in cyclic adenosine monophosphate (cAMP), with subsequent protein kinase A activation via the adenylyl cyclase (AC) pathway. The rising cAMP level leads to impaired phosphoinositide hydrolysis, the attenuation of cytosolic Ca^2+^ increase in response to agonists, and an accelerated uptake of Ca^2+^ into DTS. cAMP is an important inhibitor of platelet activation, keeping platelets in a quiescent state in circulation [137]. NO is synthetized by NO synthase (NOS) in the endothelial cells at the site of shear stress, or in the platelets by agonist action (thrombin or ADP) in the presence of increased cytosolic Ca^2+^ [142]. NO induces a rise in cyclic guanosine monophosphate via guanylate cyclase, with subsequent protein kinase G activation, which decreases the cytosolic Ca^2+^ by multiple mechanisms, causing the inhibition of platelet activation [142,143].

On this level, ADP through the P_2_Y_12_ receptor and epinephrine through its alpha 2A adrenergic receptor (α_2_A-AR) inhibit AC, reducing NO production and increasing cytosolic calcium, with subsequent stimulatory effects on platelet activation and aggregation. The P_2_Y_12_ receptor is more involved in platelet activation, granule secretion and α_IIb_β_3_ integrin activation in response to ADP, compared to the P_2_Y_1_ receptor, which has a weaker and more transient effect on platelet aggregation [141].

Platelet aggregation (3) represents the process of platelet–platelet cohesion, primarily mediated by the binding of plasma FBG to activated α_IIb_β_3_ integrin (aka GP IIb/IIIa). Each molecule of FBG binds two adjacent platelets. Following initial platelet adhesion and activation, additional platelets are recruited from the circulation to form a platelet aggregate. Platelet recruitment and α_IIb_β_3_ integrin-mediated cohesion require platelet activation by ADP, TxA_2_ release by platelet DG, and TxA_2_ generated by the adherent platelets. Thrombin is a constant contributor to platelet activation [140]. The hereafter-evolving process is complex, and further requires the tight interaction of platelets with components of the humoral coagulation system, and compounds released from the vessel wall at the site of injury, in order to maintain hemostasis.

**Figure 1:**
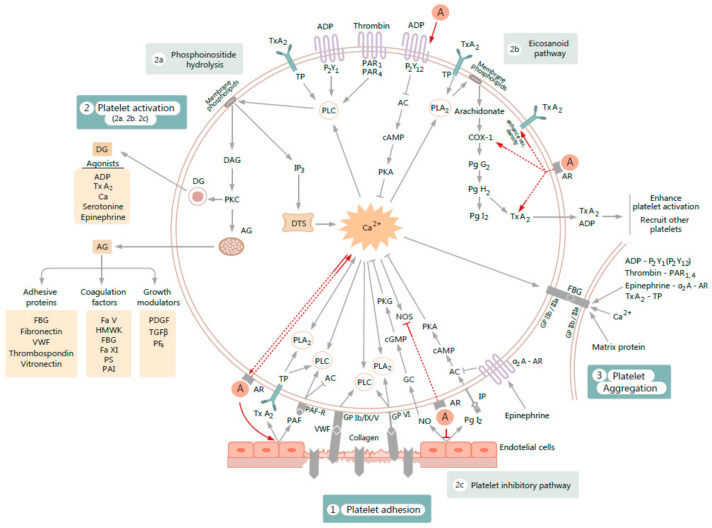
Figure illustrating the influence of exogenous androgens *A* on various processes involved in platelet activation and subsequent aggregation. The proposed mechanisms of action (figured by red arrows) are as follows: 1. *A* increase gene expression of purinergic receptor 2Y_12_ (P_2_Y_12_) of adenosine diphosphate (ADP). 2. *A* enhance cyclooxygenase-1 (COX-1) activity, increase thromboxane A_2_ (TxA_2_) synthesis, and enhance platelet TxA_2_ receptor (TP) density. 3. By acting on androgen receptor (AR), *A* enhance platelet free calcium (Ca^2+^). The rise of cytosolic Ca^2+^ increases AR affinity. 4. *A* inhibit cytosolic nitric oxide synthase (NOS) activity and generate complementary processes on endothelium by reducing nitric oxide (NO) release and suppressing prostaglandin I_2_ (PgI_2_) production. 5. *A* increase vascular TxA_2_ receptor density and endothelial TxA_2_ synthesis. 6. *A* increase platelet activator factor (PAF) activity. PAF actions on platelets involves stimulation of phospholipase C (PLC) activity and inhibition of adenylyl cyclase (AC) activity, through which PAF stimulates platelet activation and aggregation. Other abbreviations used: FBG-fibrinogen; VWF-von Willebrand factor; GP-glycoprotein; PLA_2_-phospholipase A_2_; PAF-R platelet activation factor receptor; Ca, or Ca^2+^-intracellular calcium; P_2_Y_1_-purinergic receptor 2Y_1_; PAR_1_-protease-activated receptor-1; PAR_4_-protease-activated receptor-4; IP_3_-inositol 1,4,5-trisphosphate; DTS-dense tubular system; DAG -1,2-diacylglycerol; PKC-protein kinase C; DG-dense granule; AG-alfa-granule; IP-prostacyclin receptor; GC-guanylate cyclase; cAMP-cyclic adenosine monophosphate; cGMP-cyclic guanosine monophosphate; PKA-protein kinase A; PKG-protein kinase G; α2A-AR-alpha 2A adrenergic receptor; TGFβ-transforming growth factor beta; PDGF-platelet derived growth factor; PF4-platelet factor 4; HMWK-high-molecular-weight kininogen; Fa V-blood coagulation factor V; Fa XI-blood coagulation factor XI; PS–protein S; PAI-plasminogen activator inhibitor; PgG_2_-prostaglandin G_2_; PgH_2_-prostaglandin H_2_; rec.-receptors; other symbols: ↑-up regulation, ↓-down-regulation.

The major platelet functions described above are illustrated in Figure 1.

## 4. Influence of Exogenous Androgens on Platelet Hemostatic Activity and Thrombopoiesis

The thrombogenic effect of exogenous androgens is a consequence of them shifting the hemostatic balance towards a procoagulant state.

It has been repeatedly reported that exogenous androgens are able to influence some of the aforementioned pathways leading to enhanced platelet activity. However, beginning with the first report and until today, there have been no extensive studies addressing the influence of androgens on hemostasis in a holistic way, linking platelet activation with humoral coagulation priming. In this paper, we focused on reviewing a segment of the global field of existing data—the effects of exogenous androgens on platelet hemostatic activity and thrombopoiesis.

### 4.1. Evidence from Animal Studies

The dysregulation of both platelet function and thrombopoiesis is recognized to constitute the main cause of arterial thrombosis. Considering the mounting evidence linking AAS misuse with thrombotic events, a more focused study on the effects of androgens on platelet function is needed. The animal experiments conducted to date gathered several lines of evidence to support the stimulatory influence of exogenous androgens on platelet activity and thrombopoiesis (literature summarized in Table 2).

An enhancement of platelet activity as a consequence of androgens treatment in animals was first reported by Johnson et al. (1977) [144]. The first experiment in the study showed sex differences in platelet aggregation in rats and guinea pigs (greater in males than in females). In a second experiment, an increased ex vivo platelet aggregation in response to adenosine diphosphate (ADP, 0.1–10 μg/mL) following short-term testosterone administration (1 mg/kg) was observed in female rats. To determine whether androgens have direct effects on platelets, in the absence of other hormonal environments, a third in vitro study was performed. Testosterone (1 μg/mL) was incubated with platelet-rich plasma (PRP) from male rats, and an increased platelet sensitivity in response to ADP (0.1–1 μg/mL) was obtained. Moreover, increasing levels of testosterone (91 ng/mL to 100 ng/mL) have been found to potentiate platelet aggregation (enhanced from 18.0 ± 1.5% to 62.0 ± 8.0%), when triggered by ADP (2 μg/mL). Finally, using the same turbidimetric technique, the author set a rank scale of the in vitro effectiveness of various androgens on platelet aggregation induced by 2 μg/mL ADP, which was correlated to their androgenicity—dihydrotestosterone, testosterone, methyltestosterone, androstendione, and androsterone. The study concluded that the influence of testosterone upon platelet aggregation may provide a mechanism for enhanced thrombus formation [144]. This was in accordance with previous reports indicating the thrombogenic potential of exogenous testosterone when arterial thrombosis has been induced experimentally in several animal models (mice, rats, rabbits) [93,94].

On the other hand, Skjaerlund et al. (1983) found that neither oxandrolone nor testosterone, biweekly administered in a dose of 5 mg/kg for 2.5 weeks, influenced platelet aggregation triggered by ADP (40 μM), collagen, epinephrine (6 μM) or arachidonic acid (130 μM) in platelets from atherosclerosis-susceptible White Carneau pigeons [145]. Neither of these two drugs had an influence on the synthesis of thromboxane B_2_ (the more stable product of the pro-aggregatory thromboxane A_2_) or other prostaglandins when the platelets suspension was triggered by arachidonic acid. Nevertheless, both drugs stimulated the synthesis of the more stable product prostacyclin (6-keto-prostaglandin F_1α_) and prostaglandin E_2_ in aortic homogenate of the treated pigeons after platelets suspension incubation with arachidonic acid [145].

Several years later, an ex vivo study conducted by Rosenblum and co-workers (1987) showed that testosterone-implanted pellets (1.0 mg) enhanced platelet aggregation in male, but not in female, mice, when PRP was incubated with sodium arachidonate (0.25 mM and 0.4 mM, respectively) [96]. No response was observed after DHT (0.1 mg) pellet implantation in mice of either sex, when platelet aggregation was triggered with arachidonic acid or ADP (0.25 μM) [96].

As seminal vesicles are a target organ of androgens action, Muguruma et al. (1993) examined the effect of testosterone on platelet-activating factor (PAF) activity in the seminal vesicles of guinea pigs [146]. PAF activity decreased to 50% of the normal level at 2 weeks after castration, while further testosterone propionate administration (1 mg/day, for 1 week) increased to 136% PAF activity compared to intact animals [146].

In a fairly similar way, short-term testosterone cypionate administration (10 mg/kg, twice weekly, for 2 weeks) caused a significant reduction in the threshold concentration of the TxA_2_ analog I-BOP ([1S-1α,2β(5Z),3α(1E,3R*),4α)]-7-[-3-(3-hydroxy-4-(4′′-iodophenoxy)-1-butenyl)-7-oxabicyclo-[2.2.1]heptan-2-yl]-5-heptenoic acid), necessary to produce platelet aggregation in the PRP from male rats (0.07 ± 0.01 nM in the testosterone-exposed group versus 0.45 ± 0.16 nM in the control group). Moreover, using a radioligand binding assay, short-term testosterone treatment was shown to significantly increase TxA_2_ receptor density in platelets (42.9 ± 4.2 fmol/mg protein for the testosterone-treated group versus 25.4 ± 3.2 fmol/mg protein for controls), while the TxA_2_ receptor affinity remained unchanged. Therefore, increasing the TxA_2_ receptor density might be an important mechanism underlying the thrombogenic potential of supraphysiological androgens, acting by enhancing platelet aggregation [147].

Supporting the putative pro-aggregant action of suprapharmacological AAS, we have shown in a previous published study that high doses of nandrolone decanoate (DECA), chronically administered (10 mg/kg body weight, for 12 weeks) (a situation analogous to steroid abuse), induced a significant increase in platelet aggregation triggered by ADP (2.5 μM) in the PRP from male Wistar rats [152]. This is consistent with other findings we have reported. Using the thromboelastographic method to analyze changes in the shear elasticity of clotting blood, supraphysiological DECA administration in rats showed an increase in maximal clot strength and stability (MA), which largely depends on platelet function (platelet bonding via GP IIb/IIIa) [153].

Dehydroepiandrosterone (DHEA) is an adrenal steroid and an intermediate metabolite within the androgenic pathway (prohormone), leading to the production of both androgens and estrogens. Having a weaker androgenicity, it started to be used and promoted on the bodybuilding market as a dietary supplement for gaining strength. Both human and animal studies reported beneficial effects of DHEA, including the downregulation of platelet activity. Bednarek-Tupikowska et al. (2000) noted that DHEA has no effect on platelet aggregation (following ADP 5 μmol/mL or collagen 2 μmol/mL stimulation), but she observed an increase in platelet superoxide dismutase activity (which can modulate platelet function) when the steroid was administered 0.5% in the diet (about 0.125 g/kg/day) for 12 weeks in either healthy or hyperlipidemic rabbits [148].

Several studies showed that androgen excess also stimulates thrombopoiesis, and it could increase the thrombotic risk when it is associated with an enhanced platelet aggregation [154,155]. Testosterone propionate, administered in a dose of 10 mg every other day for 6 weeks, enhanced platelet count in New Zealand intact male rabbits, while castration had an opposite effect [149]. In another study, the castration of BALB/c mice (an albino, laboratory-bred strain of the house mouse) resulted in a decrease in erythropoiesis and thrombopoiesis (decrease in platelet count, platelet size, sulfur 35 incorporation into platelets, mean megakaryocyte ploidy, and total circulating platelet mass and count). Testosterone administration in maintenance doses for several days restored platelet production, raising the possibility that it may act on the bipotential hematopoietic precursor cell [150]. A recent trial conducted in healthy male Sprague-Dawley rats evidenced a lack of effect of testosterone propionate on platelet count, platelet aggregation (using 10 μM ADP as agonist), and thromboxane A_2_ (TxB_2_) serum level, when chronically administered in high doses (0.5 mg/kg, three times per week for 12 weeks). The author concluded that supraphysiological doses of testosterone in healthy rats (and also the right dose required to normalize plasma testosterone levels in cases of testosterone deficiency) act as a cardio-protective agent, with beneficial effects on hemostasis by enhancing the fibrinolytic activity and eliciting hypocoagulation [151].

In physiological administration, androgens seem to exert an inhibiting effect on platelet activity. DHT replacement in castrated male rats (at a dose of 0.25 mg/rat, for 2 weeks, which restored the serum DHT to physiological level) exhibited a suppression of ADP-induced platelet aggregation and adhesion, H_2_O_2_-induced platelet aggregation and TxA_2_ release from platelets, and induced a significant decrease in plasmatic TxB_2_/6-keto-PGF_1α_ ratio. All androgen effects were reversed by flutamide administration. As physiological levels of testosterone also inhibited experimental arterial thrombosis in rats, it was concluded that all these effects might be ascribed to the complex androgen modulation of platelet activation [97,156]. Another group of researchers published several in vitro studies showing that the pretreatment of endothelial cell cultures from rats with testosterone at physiological concentrations completely abolished the increased platelet adhesion induced by lipopolysaccharide proinflammatory stimulus, inhibited the ADP-induced platelet aggregation in PRP incubated with endothelial cells, and stimulated the endothelial cell growth, in a releasing NO-dependent manner. It has been suggested that the antiaggregatory action of androgens at physiological levels could be dependent on NO release from the endothelial cells. As both the modulation of vasodilators synthesis and platelet function are key processes of atherosclerosis, serum physiological testosterone may help in preventing atherosclerotic degeneration [157,158,159].

We can conclude that animal studies provide evidence to a certain extent about the putative role of supraphysiological androgens in enhancing platelet hemostatic activity and thrombopoiesis. However, the lack of more comprehensive data, the multitude of limitations in study design, the relative scarcity of underlying mechanisms, and even the contradictory findings related to various androgens formulations, make it difficult to translate this overall negative influence of exogenous androgens on platelet function and count to a procoagulant state induced by these steroids, especially in terms of thrombotic risk.

### 4.2. Evidence from Human Studies

A considerable number of studies have been developed so far on the influence of exogenous androgens on human platelets. Most data come from in vitro studies, observational studies, and from prospective studies in patients suffering from a chronic certain disease, and thus assume that they followed a long-term steroid regimen. Unfortunately, few cross-sectional studies exploring platelet activity in AAS-abusing athletes have been achieved, and for ethical reasons, there are far fewer prospective studies in healthy men (most, if not all, with short-term AAS administration).

For easier reading, a split based on studies using in vitro and ex vivo platelet function testing has been established. The representative articles have been summarized in Table 3.

#### 4.2.1. Studies Using In Vitro Platelet Function Assay

Compared to ex vivo studies, the in vitro human studies use platelets from blood samples harvested from healthy donors, or cellular lineage, which are incubated with exogenous androgens, thus eluding the ethical implications linked to their administration in humans.

Johnson and colleagues were the first to publish a human study testing platelet aggregation after incubation with androgens (Nature, 1975) [160]. Once they detected sex differences in platelet sensitivity to aggregating stimuli in the ex vivo part of the study (which was greater in females than in males, in contrast with the data coming from the experiment conducted in animals), they further investigated the in vitro platelets’ responsiveness to various agonists (ADP 1 μg/mL, adrenaline 10 μM, collagen 30 μL, arachidonic acid 1 mM). An increased platelet aggregation was detected when testosterone in a dose of 1µg/mL was incubated with human PRP [160].

Later, Pilo et al. (1981) demonstrated that testosterone can increase the ionophore A23187-inducing platelet aggregation in human washed platelets and PRP, in a dose- and time-dependent manner, accompanied by concomitant TxA_2_ and other prostaglandin products generation [161]. A more recent study showed that testosterone in concentrations not normally exerting any appreciably acute effects per se (0.75 μM, or 1.5 μM) is capable of potentiating cocaine’s effect on platelet function, in terms of enhancing TxB_2_ release (the stable metabolite of TxA_2_) and augmenting the aggregation response to arachidonate (150–200 µg/mL) and collagen (5 µg/mL for 0.75 μM testosterone, and 1–5 µg/mL for 1.5 μM testosterone). The author concluded that testosterone may supplementarily increase thrombotic risk when concomitantly used with cocaine [162].

On the other hand, androgens have been noted to interfere with platelet NO production. The pretreatment of healthy male PRP with testosterone (40 nM) led to a reduction in platelet NO level, which resulted in an increased synthesis of the pro-aggregant prostaglandin TxA_2_ and increased platelet aggregation, when triggered with 2 μM ADP. This effect has not been shown in platelets from female subjects incubated with testosterone. The inhibition of the cytosolic NOSactivity by testosterone, leading to the reduction of platelet NO level, was found to occur only in platelets from male subjects, suggesting a unique sex-specific effect of testosterone. This might explain the increased occurrence of acute coronary syndrome (ACS) in males. This also suggests that flutamide (a testosterone receptor blocker) might decrease the incidence of ACS in males suffering from prostate cancer [163].

In another in vitro study, testosterone incubated with a human megakaryocytic DAMI cells showed an upregulation of androgen receptors (AR) expression at 1, 5 and 10 nmol/L concentrations, followed by a downregulation of AR expression at 100 nmol/L concentration (DAMI cells are a cellular lineage generated ex vivo from normal human CD34(+) stem cells, often used instead of platelets that do not have nuclei, in order to study the molecular regulation of gene expression by an exogenous agents) [177]. In the same megakaryocytic cellular line, testosterone (50, 150, 450 nM) has been proven to regulate in a dose-dependent manner the gene expression and protein level of P_2_Y1_2_, which is an important G protein-coupled receptor involved in ADP-induced platelet aggregation. Conversely, no effect has been recorded after the treatment of this cellular lineage with 17β-estradiol [164].

It has also been shown that testosterone (200 nM) and dihydrotestosterone (75, 100, 200 nM) increased the TxA_2_/prostaglandin H_2_ (PGH_2_) receptor density, but did not change the receptor affinity in human erythroleukemia (HEL) cells, an effect identified to be mediated through AR, and which might be the result of the new TxA_2_/PGH_2_ receptors’ synthesis by HEL cells [165,166]. Another important finding emerging from this study was the association between increased TxA_2_ receptor density and intracellular signaling following TxA_2_ receptor binding. Testosterone induced an enhancement of intracellular free calcium in HEL cells, triggered by TxA_2_/PGH_2_ agonists (I-BOP or U-46619) [165]. As platelets have been shown to convert androstenedione to testosterone, the same research group resumed the experiments several years later and demonstrated that androstenedione, in an in vitro dose of 250, 500 or 750 nM, is able to increase the maximum number of TxA_2_-binding sites in HEL cells in response to agonist I-BOP, an effect completely antagonized by receptor antagonist hydroxyflutamide (2.5 µM, when incubated with 500 nM androstenedione). This finding raised the possibility that adrenal androgen regulates TxA_2_ receptor expression either on its own, or via conversion to testosterone, an effect mediated through AR [167].

DHEAS (DHEA-sulfated, the main form in which DHEA circulates in blood) administered in vitro (0.075, 0.15, or 0.3 mM) revealed a dose- and time-dependent inhibition of arachidonate-induced platelet aggregation in human pooled PRP, as well as a reduction in platelet TxB_2_ synthesis [168]. Similarly, DHEAS inhibited platelet aggregation at either a physiological dose (0.068 × 10^−4^ M), when triggered by thrombin (0.05, 0.025, and 0.02 U/mL), or a supraphysiological dose (3 × 10^−4^ M) when triggered by collagen (2 × 10^−6^ g/mL, aggregation decreased by 63.34 ± 9.38%), thrombin (0.05 U/mL, aggregation decreased by 64.58 ± 19.96%), and TxA_2_ analog U-46619 (1 × 10^−6^ M, aggregation decreased by 55.56 ± 18.73%). Interestingly, DHEA at either a physiological concentration (1 × 10^−4^ M) or a 44-fold higher concentration presented no effect on platelet aggregation, when stimulated with 0.05 U/mL thrombin. Additionally, DHEA at a physiological concentration did not inhibit platelet aggregation following stimulation with 2 × 10^−6^ g/mL collagen and 1 × 10^−6^ M U-46619. The author speculated that such a variability in DHEAS and DHEA’s effect on platelet aggregation may be ascribed to the difference in the platelet membrane’s permeability to these compounds. In the same study, it has been shown that DHEAS inhibits platelet dense granule secretion and protein phosphorylation caused by thrombin, therefore playing a major role in the inhibition of ADP secretion. Moreover, both DHEA and DHEAS are able to activate the NOS/cGMP/PKG (nitric oxide synthase/cyclic guanosine monophosphate/protein kinase G) pathway in human platelets [169]. Concomitantly, another study demonstrated that DHEA (100 nmol/L) prevented ADP (10 μmol/L)-induced platelet aggregation by 40% compared with controls, increased platelet NO production by 63%, increased the levels of phosphorylated endothelial NOS, and increased platelet cGMP production, when incubated with PRP from postmenopausal women with type 2 diabetes mellitus. These effects have been associated with the activation of the platelet PKCδ/eNOS/NO/cGMP (protein kinase C-delta/endothelial NOS/NO/cGMP) pathway [170].

Probably one of the most challenging assumptions that has emerged in recent decades, regarding the effect of androgens on platelet activity, is that platelets are able to uptake and convert sex steroids to their active compounds, or even to generate them from cholesterol under pathological conditions. In 2008, Sarabia et al. noted that platelets from healthy men predominantly deposit testosterone, while platelets from healthy premenopausal women predominantly deposit estradiol [178]. They evidenced a tenfold greater plasma testosterone level, and also a more than threefold greater platelet testosterone level, in males than in premenopausal or postmenopausal women [178]. It has been previously proven that human platelets express both androgen and estrogen receptors, as well as the steroidogenic dehydrogenases involved in testosterone production, being able to transform androstenedione into potent androgens [147,179,180,181,182]. Furthermore, in 2012, Garrido et al. demonstrated that human platelets can import DHEAS or estrone-sulfate and convert it to DHEA (the potential precursor used for the synthesis of sex steroids) and 17β-estradiol, suggesting an intracrine capacity of human platelets to “produce” sex hormones [183]. Recently, a trial conducted by Zaslavsky et al. identified a fully intact pathway for testosterone de novo biosynthesis from cholesterol in platelets from patients with castration-resistant prostate cancer and resistance to androgen deprivation therapy [184]. Additionally, a pool of platelet-derived androgens from these patients was proven to be sufficient to induce androgen receptor signaling in prostate neoplastic cells, leading to the growth of prostate cancer. The authors concluded that the novel paracrine mechanism of testosterone synthesis in platelets to functionally relevant levels may explain the castration-resistant status of prostate cancer, and the relapse after androgen-ablative therapies, therefore suggesting a new therapeutic target centered on extragonadal androgen biosynthesis [184].

#### 4.2.2. Studies Using Ex Vivo Platelet Function Assay

The earliest cross-sectional study regarding the effects of AAS abuse on platelet function was conducted by Ferenchick et al. (1992) in 28 recruited weightlifters [29]. A statistically nonsignificant trend toward increased platelet count and aggregation induced by ADP was registered (the agonist threshold concentration required to produce platelet aggregation in AAS users versus nonusers was 2.50 ± 0.21 µM/mL versus 2.90 ± 1.10 µM/mL). However, an association between age and increased platelet responsiveness to collagen was noted in a subgroup analysis (androgen users older than 22 years old had a significantly lower collagen threshold concentration, compared to those under 22 years old; 1.47 µg/mL versus 3.35 µg/mL). The study had several limitations (unexpected findings on urine screens for the presence of exogenous androgens in declared nonusers, heterogeneous AAS self-administration with an average of three separate androgens for each declared user, and a low number of controls) probably explaining the contradictory results. The author concluded that these findings may, however, be useful to explain the previous reported thrombotic disease in the population of AAS users [29].

Later, in a double-blind, randomized and placebo-controlled study conducted by Ajayi et al. (1995) in 16 healthy men, 200 mg of intramuscular testosterone cypionate, given twice 2 weeks apart, determined a significant increase in maximum platelet aggregation (5.2 ± 1.6% at 2 weeks, a peak effect of +7.3 ± 2.3% at 4 weeks, and a return to pretreatment baseline of −0.44 ± 3.1% at 8 weeks) in response to thromboxane analog I-BOP (0.25 to 100 nmol/L) [83]. Testosterone treatment also evidenced a significant increase in platelet TxA_2_ receptor density (0.95 ± 0.13 at baseline, 1.51 ± 0.22 at 2 weeks, 2.10 ± 0.43 at 4 weeks, and 1.10 ± 0.15 at 8 weeks) in response to thromboxane analog [^125^I]BOP (80 µl, 40,000 cpm), which correlated with the endogenous testosterone concentrations of the enrolled men [83]. Additionally, the same research group demonstrated in a cross-sectional case-control study that androgen receptor blockade, or the inhibition of testosterone production by surgical and/or medical castration of men with prostate cancer, downregulated the platelet TxA_2_ receptor density, and also reduced platelet aggregation [185]. These findings let the authors conclude that testosterone may act as a regulator of human platelet TxA_2_ receptors’ expression, and this may contribute to the thrombogenicity of AAS.

Conversely, Khan et al. (2006) identified no influence of oxandrolone on ADP-induced platelet aggregation in short-term administration (intake of 10 mg twice daily for 2 weeks) in 14 healthy subjects [171]. The lack of effect has been linked to platelets’ TxA_2_ synthesis inhibition, induced by factor V and X elevation, as has been previously demonstrated by the same group of authors [186].

In a prospective randomized, double-blind, placebo-controlled study, the oral administration of 300 mg of DHEA three times a day for 14 days in healthy men showed a delay in platelet aggregation when the platelets suspension was triggered by arachidonic acid. However, the study presents important limitations [168]. In elderly subjects, physiological doses (50 mg p.o. daily) and long-term administration (for 2 months) of DHEA induced an increase in platelet cGMP levels, as a marker of nitric oxide production, which may suggest a possible antiatherogenic effect of this steroid in physiological concentrations [187].

It has also been shown that testosterone and its reduced form dihydrotestosterone (DHT) have an inhibitory effect on platelet function in physiological concentrations. In a recent study assessing platelet reactivity by whole blood impedance aggregometry (in response to arachidonate, ADP, or collagen), and by using flow cytometry to measure platelet GP IIb/IIIa complex and P-selectin expression, a negative association between these parameters and the plasma concentrations of testosterone and DHT from older people was reported (the ex vivo part of the study). The data were later confirmed by the second, in vitro part of the study, which demonstrated that testosterone and DHT in progressively increased concentrations are able to inhibit platelet aggregation in response to collagen and arachidonic acid [188].

Substantial evidence documented the stimulatory influence of exogenous androgens on thrombopoiesis. In a large-scale retrospective study assessing the long-term benefits and side effects of the weak androgen danazol, prescribed for primary immune thrombocytopenia (ITP) in 103 patients, treatment with low to medium doses (200 or 300 mg daily) for a median duration of therapy of 7 months resulted in an enhancement of platelet count from 220 × 10^9^ (/L) to 500 × 10^9^ (/L) [172]. Additionally, in a recent multicenter, randomized, open-label, phase 2 trial, combined oral therapy with danazol (200 mg twice daily, for 16 weeks) and all-trans retinoic acid was shown to be safe and effective in adult patients with ITP resistant to corticosteroid therapy [189]. The beneficial effect of danazol has also been shown in treating aplastic anemia and myelodysplastic syndromes (MDS), characterized by ineffective hematopoiesis and variable degrees of peripheral cytopenia [190,191,192]. A retrospective study has recently indicated that danazol in a median dose of 400 mg per day, for a median follow-up of 12 months, could be considered as the first-line therapy in patients with MDS who cannot receive allogeneic stem cell transplantation [173].

There are also several cross-sectional studies indicating an enhancement of thrombopoiesis consecutive to long-term AAS abuse in bodybuilders, powerlifters, or athletes [174,175,193]. The platelet count appears to return to normal after at least 1 year of androgens withdrawal [193].

On the other hand, no effect on platelet count has been reported in healthy men subjected to testosterone undecanoate administration (1000 mg given in study weeks 0, 6, 12, and 18) [176]. The lack of effect on platelet count is probably just a delay, as the hematopoietic effect of androgens does not seem to peak until 5 months [194].

In patients with polycystic ovary syndrome (PCOS) phenotypes 1 and 2 (linked with hyperandrogenemia), an increase in platelet-derived microparticles (PMPs) has been noted, PMPs appearing to be associated with atherosclerosis and also with a procoagulant profile [195,196]. The high mean platelet volume (MPV) has been recently suggested as a new indicator of cardiovascular disease risk. Studies on PCOS with elevated androgen levels tried to verify if any correlations between the levels of serum MPV and free testosterone could be evidenced, but findings are contradictory [197,198].

Overall, human studies bring more consistent evidence than experimental ones regarding the effects of exogenous androgens on platelet function and thrombopoiesis. Although several formulations, such as DHEA, or its sulfated form DHEAS, repeatedly indicated an inhibitory action on platelet function, most other types of androgens generally had a detrimental influence on platelet hemostatic activity and count, proven by both in vitro and ex vivo studies. Nevertheless, the scientific support for their prothrombotic effects is not compelling enough, and the present findings must be substantiated in further human clinical trials, other than classical observational studies on AAS abuse, or prospective ones conducted in patients subjected to prolonged androgen treatments for various diseases. Moreover, the intimate mechanisms need further clarification so as to provide a basis for bold conclusions.

Exogenous androgens lead to platelet activation and subsequent aggregation probably because of the concertedly operated nongenomic and genomic effects. Still far from being completely characterized, the main molecular mechanisms may be summarized in several distinct pathways (Figure 1), such as the following: enhancement of platelet cyclooxygenase activity [161,199,200]; increase in platelet TxA_2_ receptor density and TxA_2_ synthesis (usually determined in TxB_2_ form) [83,147,161,162,163], followed by an enhanced intracellular signaling through TXA_2_-receptor binding, which in turn results in an increased level of platelet free calcium [201]. This effect was identified to be mediated through AR and might be the result of the synthesis of new TxA_2_/PGH_2_ receptors [165,166], also leading to an increase in intracellular AR affinity for testosterone [165,180,202]. Androgens are also able to increase PAF activity [146], to induce the diminution of platelet NO level (through the inhibition of the platelet NOS activity) [163], and to generate complementary processes on the endothelium that may lead to platelet activation, such as a reduction in NO release [112,113,114,124], the suppression of PgI_2_ production [203,204], and an increase in vascular TxA_2_ receptor density and endothelial TxA_2_ synthesis [147,205]. Moreover, exogenous androgens have also been proven to increase the in vitro gene expression of the ADP receptor P_2_Y_12_ in human megakaryocytic DAMI cells [164], and to increase TxA_2_ receptor density and intracellular free calcium in HEL cells, which may be also linked to platelet activation [165,166,167].

## 5. Conclusions

In summary, the evidence presented herein leads to the conclusion that AAS excess may generate a prothrombotic state based on elevated platelet count, platelet agonists reactivity, and platelet activation with subsequent enhanced platelet aggregation. As noted, most of the reviewed human and animal studies revealed a stimulatory action of exogenous androgens on platelet function and count, whereas only a few others indicated a neutral, or even an inhibitory, effect. Regarding the influence of androgens in physiological doses on platelet function, researchers agreed on them having a beneficial effect. The heterogeneity of the findings results from imponderable factors, such as the various types of androgens used, the large variability in doses, time of intake, way of administration, or cycle of drug-use in athletes, as well as the variable number of subjects included in studies, the hemostatic status of the human subjects prior to treatment, or even their individual susceptibility to androgen intake or therapy.

Even if there is a comprehensive body of evidence suggesting the existence of a pro-aggregant effect of exogenous androgens, the link between AAS abuse and thrombosis remains to be more clearly established. This causal relationship must also refer to the influence of androgen excess over the humoral system of coagulation and fibrinolysis, another subject of debate. The gathered data in this area suggest that AAS abuse induces an overactivation of the hemostatic system, with both procoagulant and fibrinolytic effects [104]. Depending on various exogenous or endogenous factors, at a certain moment the procoagulant action probably overcomes the fibrinolytic one. The coagulation abnormalities, especially when associated with increased platelet activity, may therefore lead to a thrombotic diathesis, which could explain the multitude of thromboembolic events reported so far in the AAS-abusing population.

Despite over fifty years of research on the thrombotic risk of androgens, the current state of knowledge in this regard is still scarce. Taking into account the fact that the prospective longitudinal studies in humans have unavoidable limitations, because of obvious ethical considerations, it is a certainty that at least more epidemiological studies with thrombosis outcome, and animal experiments with “real-life” AAS dosages are warranted, in order to elucidate the overall effect of supraphysiological androgens on hemostasis and to provide new compelling evidence for their claimed thrombogenic potential.

## Figures and Tables

**Table 1:** Main characteristics of selected androgens used in diseases and clinical conditions, or abused by athletes (androgenic: anabolic ratio is that according to Kuhn C.M. et al. 2002 [2]).

Types of Androgens	Structural Classification	Way of Administration	Androgenic: Anabolic Ratio
Stanozol	17-α derivative	Oral, parenteral	1:30
Oxandrolone	17-α derivative	oral	1:10
Nandrolone	17-β derivative	parenteral	1:10
Oxymetholone	17-α derivative	oral	1:9
Methandrostenolone	17-α derivative	oral	1:5→1:2
Methyltestosterone	17-α derivative	oral	1
Testosterone			1

**Table 2:** The characteristics of included studies.

Reference	Treated Subjects, Sex, Number of Subjects Per Group, Type of Experiment	Androgen Formulation	Design of the Study	Platelet Aggregation Variation (Agonist)	Outcome from Other Assays of Platelet Function	Platelet Count
Johnson M. 1977 et al. [144]	Rats, females, *n* = 8, ex vivo	Testosterone	1 mg/kg, s.c, single administration	↑(ADP-0.1–1 μg/mL)		
	Rats, males, *n* = 8, in vitro	Testosterone	1 μg/mL, 30 min before adding ADP	↑(ADP-0.1–1 μg/mL)		
Skjaerlund JM. 1983 et al. [145]	Pigeons, females, *n* = 6, ex vivo	Testosterone Oxandrolone	5 mg/kg, i.m, biweekly, for 2.5 weeks	Neutral (ADP-40 μM, collagen, epinephr-6 μM, arach. ac. -130 μM)	Neutral effect on platelet TxB_2_, PG F_2α_, PG E_2_ synthesis from [^14^C] arach. ac. (40 mCi/mM)	
Rosenblum WI. 1987 et al. [96]	Mice, *n* = 10, ex vivo	Testosterone	Pellets, 1.0 mg, s.c, 8–19 days before the experiment	↑(arach. ac, 0.25 mM -male mice) Neutral (arach.ac, 0.4 mM -female mice)		
	Mice, *n* = 11, ex vivo	DHT	Pellets, 0.1 mg, s.c, 8–19 days before the experiment	Neutral (arach. ac, ADP -0.25 μM) -male and female mice		
Muguruma K. 1993 et al. [146]	Guinea pigs, castrated males, ex vivo	Testosterone propionate	1 mg/day, i.m, for 1 week	↑(PAF solution, 3 × 10^−11^ M, 5 × 10^−11^ M, 7 × 10^−11^ M -prepared from seminal vesicles of guinea pig added to washed rabbit platelets)		
Matsuda K. 1994 et al. [147]	Rats, males, *n* = 7, ex vivo	Testosterone cypionate	10 mg/kg, i.m, twice weekly, for 2 weeks	↑(TC for I-BOP in A vs. C group: 0.07 + 0.01 nM vs. 0.45 + 0.16 nM)		
	Rats, males, *n* = 15, ex vivo	Testosterone cypionate	10 mg/kg, i.m, twice weekly, for 2 weeks		↑B_max_ (80 μL of [^125^I]BOP)	
Bednarek-Tupikowska G. 2000 et al. [148]	Rabbits, male, *n* = 11, ex vivo	DHEA	0.5% of DHEA added to diet (0.125 g/kg/day), for 12 weeks	Neutral (ADP-5 μmol/mL, collagen-2 μmol/mL)		
Aydilek N. 2005 et al. [149]	Rabbits, males, *n* = 8, ex vivo	Testosterone propionate	10 mg, s.c, every other day, for 6 weeks			↑
Sullivan PS. 1995 et al. [150]	Mice, castrated males, ex vivo	Testosterone propionate	Maintenance doses, for several days			↑
Alhawiti NM. 2018 et al. [151]	Rats, males, *n* = 10, ex vivo	Testosterone propionate	0.5 mg/kg, three times per week, for 12 weeks	Neutral (ADP-10 μM)		Neutral
Roşca A. 2013 et al. [152]	Rats, males, *n* = 10, ex vivo	DECA	10 mg/kg, i.m, weekly, for 3 months	↑(ADP-2.5 µM)		
Roşca A. 2013 et al. [153]	Rats, males, *n* = 10, ex vivo	DECA	10 mg/kg, i.m, weekly, for 3 months		↑Maximal clot strength and stability	

Abbreviations: *n*-number of subjects per group; ADP-adenosine diphosphate; epinephr-epinephrine; arach. ac-arachidonic acid; TxB_2_-thromboxane B_2_; PG F_2α_-Prostaglandin F_2α_; PG E_2_-Prostaglandin E_2_; [^14^C] arach. ac - arachidonic acid labeled with radioactive carbon-14; DHT-dihydrotestosterone; PAF-platelet activation factor; TC-the agonist threshold concentration required to produce platelet aggregation; I-BOP-[1S-1α,2β(5Z),3α(1E,3R*),4α)] -7-[-3-(3-hydroxy-4-(4′′-iodophenoxy)-1-butenyl)-7-oxabicyclo-[2.2.1]heptan-2-yl]-5-heptenoic acid; A-androgen; C-control; B_max_-platelet thromboxane A_2_ receptor density; [^125^I]BOP-radioactive I-BOP; DHEA-Dehydroepiandrosterone; DECA-nandrolone decanoate; other symbols: ↑-up regulation, ↓-down-regulation.

**Table 3:** The characteristics of included studies.

Reference	Human subjects, Number of Individuals Per Group	Androgen Formulation	Design of the Study	Platelet Aggregation Variation (Agonist)	Outcome from Other Assays of Platelet Function, or from Tests Involving Cellular Lineage (Agonist)	Platelet Count
**In vitro**						
Johnson M. 1975 et al. [160]	Healthy men and women	Testosterone	1 μg/mL, 30 min before adding agonist	↑(ADP-1 μg/mL, adrenaline-10 μM, collagen-30 μL, arach. ac-1 mM)		
Pilo R. 1981 et al. [161]	Healthy subjects	Testosterone	Variable doses	↑(ionophore)-in a dose and time dependent manner	↑platelet TxA_2_ synthesis (and other PG products)	
Togna GI. 2003 et al. [162]	Healthy volunteers	Testosterone and cocaine	- Testosterone 0.75 μM and cocaine 50 or 100 μM, 10 min - Testosterone 1.5 μM and cocaine 100 μM, 10 min	- Neutral (collagen-1–3 µg/mL), ↑(arach. ac-150–200 µM) - ↑(collagen-1–5 µg/mL, arach. ac-150–400 µg/mL)	- Neutral on platelet TxB_2_ production (collagen-1–3 µg/mL); ↑ platelet TxB_2_ production (arach. ac-150–200 µM) - ↑platelet TxB_2_ production (collagen-1–5 µg/mL, arach. ac-150–400 µg/mL)	
Banerjee D. 2014 et al. [163]	- Healthy male volunteers - Healthy female volunteers	- Testosterone - Testosterone	- 40 nM, 40 min - 40 nM, 40 min	- ↑(ADP-2 μM) - Neutral (ADP-2 μM)	- ↓ platelet NO, ↑ platelet TxA_2_ synthesis (ADP-2 μM); - Neutral on platelet NO, and TxA_2_ synthesis (ADP-2 μM)	
Lee SJ. 2012 et al. [164]	Human megakaryocytic DAMI cell line	Testosterone	50 nM, 150 nM, 450 nM, for 36 h.		↑P_2_Y_12_ mRNA and P_2_Y_12_ protein level in a dose-dependent manner	
Matsuda K. 1993 et al. [165], Halushka PV. 1994 et al. [166]	Human erythroleukemia cells	- Testosterone - DHT	- 200 nM, for 24 h - 75, 100, 200 nM, for 24 h		↑B_max_ ([^125^I]BOP-50 pM) following T and DHT administration; ↑[Ca^2+^]_i_ ([^125^I]BOP-100 nM, or U-46619) for T administration	
Zucker TP. 1996 et al. [167]	Human erythroleukemia cells	- Testosterone; - Androstenedione	- 150 nM, for 48 h; - 250, 500 or 750 nM, for 48 h		↑B_max_ ([^125^I]BOP-60 pM)	
Jesse RL. 1995 et al. [168]	Healthy donors	DHEAS	0.075, 0.15, or 0.3 mM, for 1 min	↓(arachidonic acid)	↓TxB_2_ synthesis (arachidonic acid)	
Bertoni A. 2012 et al. [169]	Healthy donors	DHEAS	- 0.068 × 10^−4^ M, for 1 min; - 3 × 10^−4^ M, for 1 min	- ↓(thrombin-0.05 U/mL, 0.025 U/mL, 0.02 U/mL) - ↓(collagen-2 × 10^−6^ g/mL, thrombin- 0.05 U/mL, U46619–1 × 10^−6^ M)	Activation of platelet NOS/cGMP/PKG pathway (DHEAS at 3 × 10^−4^ M)	
Munoz YC. 2012 et al. [170]	Postmenopausal women, type II diabetes mellitus	DHEA	100 nmol/L, for 20 min	↓(ADP-10 μmol/L)	Activation of platelet PKCδ/eNOS/NO/cGMP pathway	
**Ex vivo**						
Ferenchick G. 1992b et al. [29]	-Weightlifters, *n* = 24 for A users; -Weightlifters, *n* = 13 for A users, stratified by age	Various type AAS intake (an average of three separate AAS/each user)	Various doses and length of AAS use	- Neutral [TC in AU vs. N group: 2.50 ± 0.21 µM/mL vs. 2.90 ± 1.10 µM/mL (for ADP); 2.50 ± 0.38 µg/mL vs. 1.96 ± 1.11 µg/mL (for collagen)]; - ↑(age subgroup analysis, TC for collagen in AU > 22 yo vs. AU ≤ 22 yo group: 1.47 µg/mL vs. 3.35 µg/mL)		Neutral
Ajayi AA. 1995 et al. [83]	Healthy men, *n* = 9	Testosterone cypionate	200 mg, i.m, given twice, 2 weeks apart	- ↑(TxA_2_ analog I-BOP-0.25 to 100 nmol/L) - Neutral (thrombin-0.00625 to 0.1 U/mL)	↑B_max_ ([^125^I]BOP-80 µl);	
Kahn NN. 2006 et al. [171]	Healthy subjects, *n* = 14	Oxandrolone	10 mg, twice daily, for 2 weeks	Neutral (ADP)		
Jesse RL. 1995 et al. [168]	Healthy men, *n* = 5	DHEA	300 mg, p.o, 3 times daily, for 14 days	↓(arachidonic acid)		
Liu W. 2016 et al. [172]	Patients with ITP, *n* = 103	Danazol	200 or 300 mg daily, for a median duration of 7 months			↑
Colunga-Pedraza PR. 2018 et al. [173]	Patients with MDS, *n* = 42	Danazol	Median dose of 400 mg/day, median follow-up of 12 months			↑
	Bodybuilders and powerlifters, *n* = 17 (AAS abusers); *n* = 15 (AAS ex-abusers)	Heterogenous AAS intake	Mean dosage of 750 mg/week, 33 weeks per year, over 8 years (AAS abusers); mean dosage 700 mg/week, for 26 weeks per year, over 9 years (AAS ex-abusers)			↑
Ansell JE. 1993 et al. [174]	Bodybuilders, *n* = 11	Various type of AAS intake, 3 different AAS/each user (average)	Various doses and length of AAS intake			↑
Severo CB. 2013 et al. [175]	Weightlifters, *n* = 10	Various type of AAS intake, 3 different AAS/each user (average)	Various doses and length of AAS intake			↑
Zitzmann M. 2002 et al. [176]	Healthy men, *n* = 14	Testosterone undecanoate	1000 mg, i.m, in study weeks 0, 6, 12, and 18			Neutral

Abbreviations: TxA_2_-thromboxane A_2_; PG-prostaglandins; NO-nitric oxide; DAMI-represents a cellular lineage generated ex vivo from normal human CD34(+) stem cells, often used instead of platelets; P_2_Y_12_ - purinergic receptor 2Y_12_; P_2_Y_12_ mRNA- purinergic receptor 2Y_12_ gene expression; DHT-dihydrotestosterone; T-testosterone; [Ca^2+^]_i_-intracellular calcium concentration; U-46619-TxA_2_/Prostaglandin H_2_ agonist; DHEAS-DHEA-sulfated; NOS/cGMP/PKG pathway-NO synthase/cyclic guanosine monophosphate/protein kinase G pathway; PKCδ/eNOS/NO/cGMP pathway-protein kinase C-delta/endothelial NOS/NO/cGMP pathway; AAS-anabolic androgenic steroids; TC-the threshold agonist concentration required to produce platelet aggregation; AU-AAS users; N-nonusers; yo-years old; ITP-Primary Immune Thrombocytopenia; MDS-myelodysplastic syndrome; ↑-up regulation, ↓-down-regulation.
